# Clustering of Codons with Rare Cognate tRNAs in Human Genes Suggests an Extra Level of Expression Regulation

**DOI:** 10.1371/journal.pgen.1000548

**Published:** 2009-07-03

**Authors:** Joanna L. Parmley, Martijn A. Huynen

**Affiliations:** Centre for Molecular and Biomolecular Informatics, Nijmegen Centre for Molecular Life Sciences, Radboud University Nijmegen Medical Centre, Nijmegen, The Netherlands; Stanford University, United States of America

## Abstract

In species with large effective population sizes, highly expressed genes tend to be encoded by codons with highly abundant cognate tRNAs to maximize translation rate. However, there has been little evidence for a similar bias of synonymous codons in highly expressed human genes. Here, we ask instead whether there is evidence for the selection for codons associated with low abundance tRNAs. Rather than averaging the codon usage of complete genes, we scan the genes for windows with deviating codon usage. We show that there is a significant over representation of human genes that contain clusters of codons with low abundance cognate tRNAs. We name these regions, which on average have a 50% reduction in the amount of cognate tRNA available compared to the remainder of the gene, RTS (rare tRNA score) clusters. We observed a significant reduction in the substitution rate between the human RTS clusters and their orthologous chimp sequence, when compared to non–RTS cluster sequences. Overall, the genes with an RTS cluster have higher tissue specificity than the non–RTS cluster genes. Furthermore, these genes are functionally enriched for transcription regulation. As genes that regulate transcription in lower eukaryotes are known to be involved in translation on demand, this suggests that the mechanism of translation level expression regulation also exists within the human genome.

## Author Summary

The degeneracy of the genetic code means that many amino acids are encoded by not one, but a range of codons. In bacteria and yeast, it is known that the choice of codons used can be beneficial (or detrimental) to the gene function. As humans have a relatively small effective population size, and the efficiency of selection to purge mutations of mild deleterious effect decreases as population size decreases, it has been assumed that the benefit/cost of codons is not large enough to have a measurable effect on codon choice. Here we show that codons with the lowest amount of tRNA are clustered in gene sequences more often than anticipated. The genes containing these clusters were found to have specific functions in gene expression. Comparisons to known bacterial and yeast processes suggest a translation level mechanism for the regulation of protein expression in human genes. Thus, our investigation highlights the potential for the presence of a novel regulatory mechanism in human genes.

## Introduction

Codon usage bias is abundant in every sequenced genome and several theories have been put forward to explain it, depending on the genome or the gene. In many organisms, including bacteria, yeast and *Drosophila* species, the strongest factor determining codon bias is selection for maximizing translation speed and accuracy [1]–[6]. Those genes that are the most highly expressed exhibit a bias towards codons that have the most abundant cognate tRNAs. It is not the case, however, that a maximal rate of translation always results in optimal protein production. In a handful of cases the synonymous mutation of a codon to the most translationally optimal will cause a phenotype [7],[8]. In bacteria and yeast there are several well-studied mechanisms by which local variations in translation rate are an essential regulator of protein production [9]–[11].

Protein secondary structure is known to be influenced by the local rate of translation and translational pausing [see 12]. The stalling of translating ribosomes can allow nascent proteins the freedom to fold, or facilitate the interaction of chaperones/regulatory proteins, without the interference from the physiochemical properties of the downstream protein sequence [see 8]. Furthermore, different protein secondary structures are associated with codons with different translation rates. For example, in *Escherichia coli*, beta strands are more commonly associated with codons with low levels of cognate tRNAs, whereas alpha helices associate with codons with abundant cognate tRNAs [12]. More generally, rare codons are found near the boundaries of protein domains [12]–[14] in *E.coli*.

Variable local translation rate is used in several species as an extension of expression level regulation. This is especially so in the case of trypanosomatids, which have little regulation of gene transcription and instead have been suggested to rely on mechanisms that influence the rate of translation to fine-tune protein levels [15]. The expression of genes can be down regulated at the translation level by a process called no-go decay (NGD). This system is thought to be a safety mechanism to clear blocked mRNAs and is characterized by the dissociation of the stalled ribosome from the mRNA, followed by the degradation of both the nascent protein product and the mRNA [see 16]. NGD allows the translation at low levels of those genes that are highly transcribed.

The presence of NGD, in turn, opens up the possibility for translation on demand, a mechanism thought to occur in *Saccharomyces cerevisiae* to minimize the reaction time to a stress stimulus. If, under normal conditions, translation is limited by ribosomes that stall at a specific mRNA position, then protein production can be rapidly up regulated in response to a stress factor by resuming translation. Regulating a response to stress via this path will elicit a faster response than if the control was solely at the level of transcription [17]. The genes most commonly regulated by translation on demand are transcription factors and those related to gene processing [18]: genes that can go on to alter the expression profile of other genes.

In humans, there has been much contradictory and inconclusive evidence for the presence of selection for translation optimization [4], [19]–[21]. There are several reasons for this lack of certainty. It is thought that selection for the purging of weakly deleterious mutations is relatively inefficient in mammals due to a limited effective population size [22]. In addition, a large component of codon bias in mammals can be explained by variations in the local GC content. There may also be a conflicting effect by purifying selection acting on exonic splicing regulatory elements, a mechanism not as prevalent in lower eukaryotes, with the potential ability to out compete any translation level selection [23],[24]. More recently however, strong evidence has been provided that optimal codons (those codons with the most abundant tRNAs) associate with conserved sites within human genes [25], prompting the proposal that there is selection to limit errors in translation of human genes.

Further recent investigations by Kimchi-Sarfaty highlighted that synonymous changes for “slow” codons can have a detrimental effect in human genes [7]. In the specific case of *MDR1* the disease phenotype is observed when a haplotype of SNPs for rare codons occur [7]. Kimchi-Sarfaty proposed that this is due to the effect of the rare codons on the translation rate, which compromises the folding of the nascent protein, thus diminishing the function of the mature protein.

**Figure 1:**
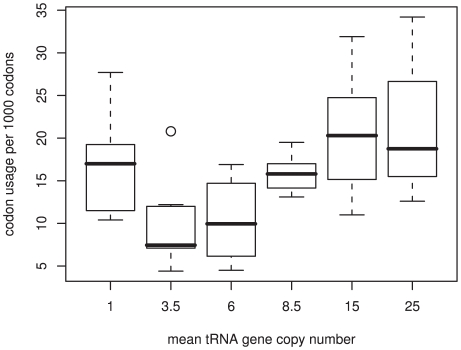
Correlation between tRNA gene copy number and genome codon usage. There is an overall trend for codons with high genome usage to have more cognate tRNA gene copies. However, the codons with the fewest cognate tRNA genes are not the most rare within the genome.

Regions of genes that may regulate the folding of mature proteins, by means of rare codon clusters, have been identified in two studies [26],[27]. Widmann *et al.* assessed the usage of rare codons in genes from two families of α/β proteins and found that synonymous mutations in these clusters induce protein mis-folding [27]. The protein families investigated were those most likely to undergo co-translational protein folding, and thus, these results do not represent the incidence of any genome-wide phenomena. Clarke and Clark proposed a large-scale method for identifying gene segments of highly biased codons (when compared to their potential maximum bias) [26]. Both these studies (mentioned above) attributed the clustering of rare codons to constraints on protein folding. However, these two investigations may suffer from the assumptions that they have made. Firstly, both groups assume that the codons used most infrequently in the genome are those that will be the least translationally optimal. There is no evidence for this. If we take the number of cognate tRNA gene copies as a proxy for the rate of translation of the codon, then codons with the fewest tRNAs, and thus the lowest rate of translation, do not have the lowest genome frequency (Figure 1). Secondly, both groups identify codon bias within the genes relative to the whole genome codon usage, and ignore the variations in local GC content across the human genome. This approach may fall foul of isochore effects in mammalian genomes.

We propose an alternate method to identify clusters of translation rate-limiting codons that may be of functional importance in human genes. This method is free from local nucleotide biases and assumptions about the usage of codons throughout the genome. Further, we assume that the largest factor determining the rate of translation of a codon is the number of cognate tRNA genes. With this approach we determine the prevalence of translation rate-limiting clusters in human genes and, without prior assumptions about their function, assess genic properties to infer the potential role of these clusters.

## Results

### Clusters of codons with low tRNA gene copy numbers are common in human genes

**Figure 2:**
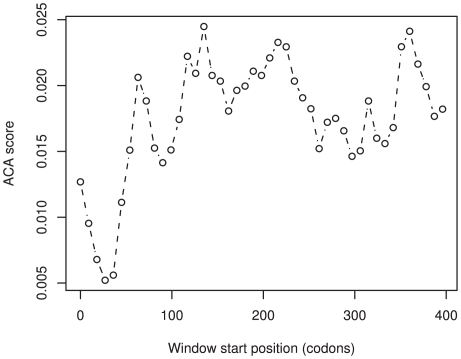
The sliding window profile of FOXF2: a lung and placenta specific transcription factor. Although there is large variation across the gene, the ACA score at the 5′ region is very unlikely to have occurred by chance (P<0.001).

**Figure 3:**
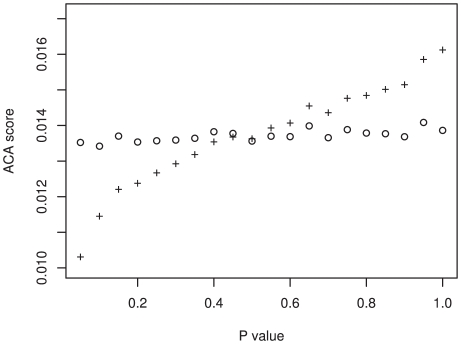
The difference between the true ACA scores and the randomized ACA scores. The difference between the randomized ACA score (hollow circles) and true ACA scores (plus sign) is displayed for all p-values. The true ACA score was deemed significantly different to the randomized ACA score if the p-value< = 0.05.

To identify regions of genes that have the greatest potential to minimize the translation rate, we devised a measurement of corresponding tRNA abundance (the anti-codon abundance score). This score assumes that there is a direct correlation between tRNA abundance and the number of tRNA genes; an assumption that previous investigations have shown is justified [2],[28],[29]. This scoring method allows us to directly compare the different amino acids within the same gene. We employed a sliding window analysis across 13,793 human genes and calculated the average anti-codon abundance score (ACA score) for each window (see Methods and Figure 2). The region of the gene with the lowest score was deemed to have the greatest putative role in the reduction of translation rate. This classification differs from other methods that found the regions of the greatest codon bias when compared to the codon usage of the whole genome, a method that does not guarantee that the region identified limits the translation rate. Our method identifies the absolute rate-limiting position within the gene, the region most likely to cause translation related regulatory effects. To test if the window with the lowest ACA score was expected given the underlying nucleotide content of the gene, or whether it occurred due to factors other than chance alone, we implemented a randomization analysis. For each gene, the existing codons were shuffled 1,000 times, maintaining the underlying gene codon usage and nucleotide biases, and the sliding window analysis was repeated. We identified 1703 genes with an original ACA score that was lower than at least 95% of the randomizations for that gene and 148 genes with an ACA score that was lower than 99.9% of the randomizations (Figure 3).

Of course, in any large dataset of genes one would expect to find a number of genes with a low ACA score. To determine our false discovery rate, we employed the QVALUE software [30],[31]. Provided with the distribution of p-values for all the genes, whether they were deemed significant or not, QVALUE will calculate the proportion of false positives that would be expected if a p-value was to be used as the significance cut-off. At our chosen significance threshold p-value of 5%, we had a false discovery rate of 23%. Thus, of our initial 1703 clusters that were found to have significantly low scores, 391 may have been falsely identified. Nevertheless, this leaves 1306 genes that are likely to be true positives. Thus, we find that up to 10% of the genes in the entire human gene set contain a region with a significantly low score. To the regions of the genes that we found to have significantly low scores we allocate the term RTS (Rare tRNA Score) clusters.

### Putative pause sites are not due to the encoded protein sequence

It is possible that some amino acids are encoded by a set of synonymous codons that all have a relatively low number of tRNA genes. If these amino acids occur together in a protein domain, we could see significant RTS clusters due to constraints at the protein level. To evaluate the impact of protein level interference, we employed a second randomization. In order to control for local nucleotide biases and isochore effects, we binned the genes into 138 groups of 100 genes of similar G/C content at third codon positions (GC3). For each randomized iteration the amino acid sequence of the RTS cluster was maintained and the codons were randomly selected, weighted by the synonymous codon usage within the GC-bin. The new ACA score was calculated and compared to the original. The cluster was deemed free of protein level interference if less than 5% of the randomizations produced a lower score, indicating that the RTS clusters are not the result of the amino acid sequence. Under these criteria, 601 genes were further purged, leaving 1102 putative translation pausing sites with a false discovery rate of 2.6% [31]. It is these genes that have a significantly low ACA score, after controlling for local nucleotide biases and interference from the amino acid sequence, for which we implemented the remaining analyses.

### RTS clusters show reduced substitution rates

If the RTS clusters we have identified are functionally important, we expect that there should be conservation of the cluster region. To this end, we calculated the number of synonymous substitutions between human and chimp orthologues. As synonymous substitutions between human and chimp orthologues are not common, the number of substitutions in concatenated RTS cluster regions were compared to those of concatenated non-cluster regions of the genes, after controlling for the potential influence of splicing effects. Since the evolution rate near splice sites is reduced due to the conservation of exonic splicing enhancer elements [24],[32],[33], we need to control for this within gene variation in the rate of evolution. We therefore focused our analysis on sequences distal to intron-exon boundaries. The orthologous human-chimp sequences were purged to contain only coding sequence that fell outside 70 nucleotides of a splice site. This cut-off has been used previously in the literature and it has been shown to contain the large majority of the regulatory elements; thus we assume that analyzing sequence outside this cut-off will control for a large amount of confounding effects [24],[34]. The expected values of synonymous substitutions between RTS clusters and non-cluster regions were calculated under the assumption that within the splice site distal sequence the substitutions should be evenly distributed. Fisher's exact test of these expected values against the number of observed substitutions reveals that RTS cluster regions show a significant decrease in the number of synonymous substitutions (57% of the expected value, 24 observed versus 42 expected synonymous substitutions, p = 0.01), indicating that the RTS clusters are conserved and are likely to be functionally important.

### A mechanistic role of a local reduction in translation rate

#### The distribution of RTS clusters is skewed toward genic extremities

**Figure 4:**
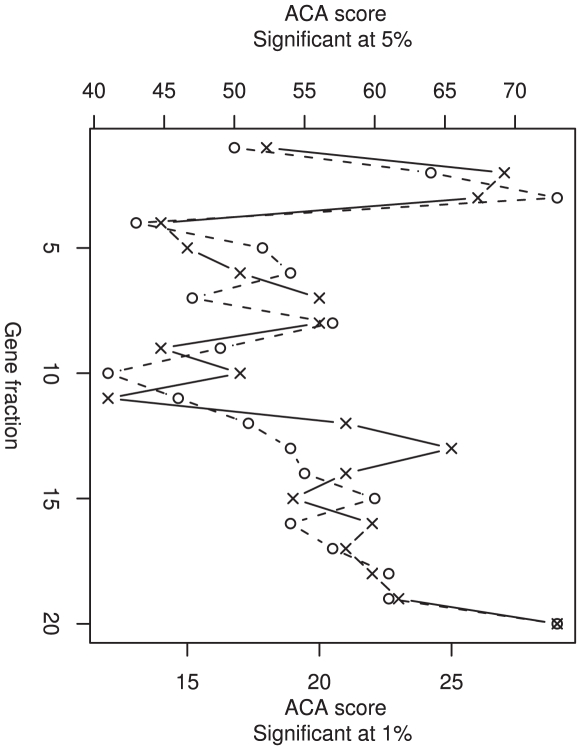
The positions of the mid-point of RTS clusters across genes. Each gene was split into 20 bins so that genes of different lengths can be compared. The positions of RTS clusters are determined by the gene fraction in which the cluster arises. The frequency of RTS clusters was not evenly distributed across genes, but skewed toward the 5′ end and the 3′ end (p<0.001). The distribution of RTS clusters defined by p<0.05 is shown by hollow circles, while those highly significant RTS clusters, defined by p<0.01, are shown by crosses.

Although the degradation of mRNA and the nascent protein associated with stalled ribosomes has been linked to the regulation of protein expression in bacteria, this “No-go decay” theory presents a major pitfall, since it would incur a large waste of resources [35]–[37]. However, if this mechanism were in place, one might expect that selection would limit such a waste. If our RTS clusters were present to facilitate NGD, we could predict that these RTS clusters would localize to the 5′ end of the gene, thus minimizing the use of resources and the size of the protein product to be degraded. To allow the comparison of genes of differing lengths, we determined in which 20^th^ of the gene the RTS cluster was located (Figure 4). We observed that there was a highly significant skew in the position of clusters towards the beginning and the end of genes (p<0.001, Chi-squared analysis), even in the most significant clusters (Figure 4). Those genes with RTS clusters skewed toward the 5′ region of the gene could be explained by the above scenario. However, selection to limit the waste of resources involved in NGD would not explain the remainder of the distribution we observe.

#### RTS cluster–containing genes have increased tissue specific expression

Recent investigations by Lavner *et al.* revealed that codon usage bias was high in highly expressed human genes; however, codon bias was higher still in genes with low expression. This is a relationship that cannot be explained by any currently known mechanisms or phenomena applied to human genomes. Lavner proposed that this finding could be due to selection for least optimal codons in those genes that have low expression to ensure low protein production [28].

To test whether our RTS cluster genes have contrasting expression profiles to the remainder of the genes, or more specifically if RTS cluster genes were expressed at unusually low levels, we compiled a dataset of mRNA expression levels across a comprehensive range of tissues (see Methods) and determined the median and maximum expression as well as the tissue specificity (TSI) of each gene (see Methods). We compared the expression profiles of RTS cluster containing genes with randomly generated gene sets of equal size from the whole gene set to discern any differences in mRNA levels. Between RTS cluster genes and random gene sets, one main difference was apparent. The tissue specificity, measured by TSI, was significantly higher in RTS cluster containing genes (p<0.001, obtained by comparison to 1,000 randomly generated gene sets), indicating that tissue specific expression is more likely in RTS cluster genes. In order to determine if this increase in tissue specificity was particular to a tissue, we developed a method to find the tissue (or few tissues) for which the expression of the gene was specific. We cannot assume that the expression levels are normally distributed, thus we determined the median and the inter-quartile range (IQR) of the expression for each gene in the whole dataset with obtainable expression data (3,133 genes). Tissues were accepted as specific for a gene if they exhibited expression greater than or equal to the median expression plus 7.5 IQRs. This method allows us to capture multiple tissues in which expression is relatively high, irrespective of overall expression level, as well as controlling for the distribution of expression values (e.g. taking as a cut-off value a multiple of the average will suffer from genes with large IQR). We compared the expected values to the observed number of RTS and non-RTS cluster genes specific for each tissue group, revealing one tissue group to be enriched in the RTS-cluster gene set. We observed a 3-fold increase in the number of RTS cluster genes specific to the brain relative to that expected by chance (p = 0.045, Fisher's exact test).

If the presence of the RTS clusters was a result of selection on genes with low expression, we would expect the average gene expression to be reduced in RTS cluster genes. As we observed no difference between the average levels of expression for RTS cluster and non-cluster genes, it is unlikely that the cause of the RTS clusters is the mechanism proposed by Lavner.

#### The RTS cluster genes show distinct transcription related GO profiles

We performed Gene Ontology analysis on those genes that contained RTS clusters to determine whether they were over-represented for any function or protein localization. This was achieved using the web-application Babelomics [38]. Comparing all genes containing RTS clusters to cluster-free genes showed an over-abundance of many linked functions that strongly suggest that our observations are biologically relevant. The strongest enrichments were observed for chromatin binding (3.15-fold increase, adjusted-p = 0.02), sequence-specific DNA binding (2-fold, adjusted-p = 0.002), transcription factor activity (1.7-fold, adjusted-p = 0.002) and RNA binding (1.65- fold, adjusted-p = 0.04). These functions are similar to those described in bacterial and lower eukaryote genes that undergo translation on demand [18].

#### There is no link between RTS clusters and protein domains

The regulation of protein folding by local variations in translation rate is the most widely researched mechanism by which translation can influence protein function in lower eukaryotes and bacteria. If there is also a link between protein folding and translation rate in human genes, we may expect a correlation between Pfam domain positions and the position of the RTS clusters, similar to those found in *E.coli*. For instance, we can speculate that it would be beneficial for translation to pause between protein domains in order for them to fold independently [13],[14],[39],[40]. To this end, we identified Pfam domains from the Ensembl database for each gene containing an RTS cluster. We classified the RTS clusters depending on whether their midpoint occurred within a Pfam domain, a flanking domain or a spacer region (see Methods). As a null, we expected the RTS clusters to be distributed evenly throughout these regions and, after Chi-squared analysis, we were unable to reject this hypothesis. There was no observable skew between the position of RTS clusters and the position of either Pfam domains or their immediate flanking regions, and thus we found no evidence for a link between protein domains and cluster presence. This observation is in agreement with that of Widmann *et al.* [27] who identified regions of rare codons within genes but found no consistency in their distribution relative to protein structure.

#### The effects of slow translation seem to be selected against in genes that undergo co-translational protein folding

It could be asked whether the above test is appropriate to assess the link between protein folding and translation rate. Recent work has argued that co-translational protein folding mainly occurs in the α/β class of proteins [41]. We would expect that if the regions of putatively low translation rate were due to a pre-requisite for protein folding regulation, then α/β proteins should be over-represented in our RTS cluster containing genes. This however was not observed. In fact, there was a significant trend for RTS clusters to be avoided in α/β proteins (p<0.05, Fisher's exact test). Thus, there is no evidence to suggest that translational pausing is necessary for the correct folding of human proteins.

### RTS clusters are not due to other confounding factors

#### RTS clusters are not due to codon usage biases near splice sites

Recent investigations into mammalian genes have revealed strong codon usage bias due to the presence of splicing regulatory elements in the exonic sequence near splice sites [23],[33]. Is it possible that the presence of these exonic splicing enhancer (ESE) sequences can cause the RTS clusters? If this were the case, we would expect to find a high density of RTS clusters within close proximity to splice sites. We examined the observed distribution of clusters in the vicinity of splice sites and the remaining gene sequence. RTS clusters are not enriched but avoided (data not shown) near splice sites, indicating that the abundance of RTS clusters is not an artifact of skewed codon usage near splice sites.

#### Genes containing RTS clusters are not associated with CpG islands

It is important to consider the potential presence of CpG islands as a source of RTS clusters, as these features are known not only to associate strongly with the 5′ region of genes, but also to encroach into the coding sequence of genes and interfere with codon usage. We assessed a dataset of 1222 CpG islands [42] to determine the observed and expected association of CpG islands with RTS cluster genes. We found no association between CpG islands and the genes that contain clusters of low score codons (data not shown).

## Discussion

Due to the relatively small effective population size of mammalian species, in addition to a lack of evidence for selection to purge weakly deleterious mutations in higher eukaryotes, it has been assumed that selection for a mechanism of gene regulation programmed within the coding sequence of mammals does not occur [43],[44].

In this investigation, we show that clusters of codons with low cognate tRNA gene copy numbers are more common than expected given local codon usage and constraints from the amino acid sequence. The potential importance of these RTS clusters is highlighted by the significant reduction in synonymous substitutions in chimp orthologues at the RTS cluster regions. Further, these observations cannot be explained by confounding factors such as CpG islands or the presence of splicing regulatory elements.

Opposed to observations in other species that beta sheet structures and the boundaries of protein domains are associated with the use of codons with low abundance cognate tRNAs [see 12],[13],[Bibr pgen.1000548-Krasheninnikov2] [14 and also 8], we observed no evidence to suggest that this occurs in humans. In fact, our RTS cluster genes are significantly underrepresented for α/β proteins, which evidence suggests are those most likely to undergo co-translational protein folding [41]. This may indicate that reduced translation rate has a negative impact on protein folding in humans, as observed in the case of *MDR1* [7].

Intriguingly, we observed two skews in RTS cluster positions within the gene: those skewed to the 5′ region and those skewed to the 3′ gene region. We also found that RTS cluster genes have higher tissue specific expression profiles than the remaining RTS cluster–free genes. Additional evidence from the Gene Ontology analysis revealed a strong over-representation of genes involved in transcription, in-keeping with those known to undergo translation on demand in prokaryotes [18].

When we take these results together, it is feasible that RTS cluster genes are subject to a process similar to NGD, a mechanism that limits the level of protein production. This potential is indicated by the fact that some clusters are skewed toward the 5′ region [see 16], a feature used to minimize the cost of employing NGD.

One alternative theory explaining the clustering of codons corresponding to rare tRNAs is one we refer to as the “recruitment delay minimization” hypothesis. The theory posits that if one rare codon is used then subsequent synonymous codons will be biased towards this codon. The reasoning is that once the tRNA has been recruited to the mRNA it will be in position to translate the proximal cognate codons without imposing a recruitment delay, and thus any impact on translation rate is minimized. As this mechanism acts to maximize the translation rate of a restricted sequence, we would expect that this bias would only be necessary in a handful of cases. If a reduction in the translation rate is costly to fitness, then selection should favor the use of synonymous codons with abundant tRNAs. The only instance where the clustering of the same slow codon to minimize recruitment delay would occur is if all the synonymous codons for an amino acid are rare. Our results are independent of this phenomenon as those RTS clusters due to amino acids with only low scoring codons were purged from our analyses. In addition, this selective force should be restricted to highly transcribed genes; a feature not enriched in our RTS cluster genes.

For the most significant RTS clusters (Table S1), site directed mutagenesis studies, which modify the nucleotide sequence to maximize translation rate, may reveal in which capacity these RTS clusters are necessary.

## Methods

### Datasets

#### Gene sequences and alignments

The human coding sequences were extracted from the refMrna.fasta file from the UCSC Genome Browser http://hgdownload.cse.ucsc.edu/goldenPath/hg18/bigZips/ [45] and orthologous chimp coding sequences were extracted from the NCBI database. In addition, the exonic boundaries were determined from the NCBI RefSeq entries for the human dataset only. The orthologous human and chimp genes were aligned at the amino acid level using muscle v3.6 [46].

#### Protein classification

For each gene, we determined whether it was classified as an α/β protein or another class by cross reference with SCOP 1.73 protein classification release [47].

#### Pfam domain mapping

The mapping of the Pfam domains to proteins was done using the database interface of the Ensembl genome browser (release 47) [48]. From this data we were able to classify RTS clusters with respect to their position in the protein structure. If the mid-point of the cluster fell within the bounds of a Pfam domain, then we assigned the cluster as a Pfam cluster. If the mid-point fell within the flanking 30 nucleotides (as long as no further Pfam domain occurred in this region) then the cluster was defined as flanking. Any remaining cluster positions were classified as spacer region clusters.

#### Expression data

The mRNA levels for human genes across 73 non-cancerous tissues were obtained from GNF Genome Informatics Applications & Datasets as Human U133A+GNF1H (gcRMA-condensed) [49], which can be accessed to see the range of tissues analyzed. Rather than use an arbitrary cut-off value to determine whether a gene was expressed in a tissue, we used the database presence/absence calls from the file GNF1h AP calls. The maximum expression level was defined as the highest observed level of mRNA measured in a tissue where the gene expression is found to be present. The median gene expression values were only calculated from those tissues where gene expression was also deemed present from these data. We assessed the tissue specificity of the expression profile of genes by employing a Tissue Specificity Index [50]. It is calculated as follows:
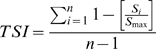
Where *S_i_* is the expression level of the *i^th^* tissue, *S_max_* is the maximum expression value and *n* is the total number of tissues. If the gene is expressed at a similar rate across a broad array of tissues then we should observe a TSI close to 0. On the other hand, if there is very high expression in only a small number of tissues then the TSI should be close to 1.

#### Sliding window analysis and the Anti-Codon Abundance Score

**Figure 5:**
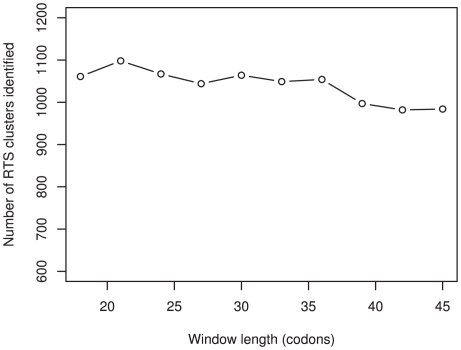
The sliding window analysis with increasing window sizes. The length of the windows employed in the sliding window analysis does not greatly affect the absolute number of significant RTS clusters identified in the dataset. However, the number of RTS clusters classified as significant decreases as the window size increases. This is expected if surrounding sequence that is not biased towards reducing the translation rate dilutes the effects of significant clusters.

A sliding window analysis was implemented on all genes. Each window was 27 codons long and was applied to every 9^th^ codon to allow ample coverage of the data, but with a conservative time constraint. To show that the results are not an artifact of this window size, the randomization was applied to the genes under varying window sizes (Figure 5). Each codon was ascribed a numerical value, calculated by dividing the number of cognate tRNA genes by the total number of tRNA genes. The tRNA gene copy numbers for human were obtained from http://lowelab.ucsc.edu/GtRNAdb/ [51]. This analysis makes the assumption that the number of tRNA genes is a true representation of the tRNA abundance within the cell. Previous studies have shown that this assumption is not unfounded [2],[28],[29]. The ACA score for each window is calculated as the mean of the codon values within that window. The window within the gene with the lowest ACA score is then defined as the potential translational pause site. Even though this definition is very crude, it is also very stringent, as only one large signal per gene will be identified. Therefore, we can be confident in our prediction of the most likely pause sites. However, multiple local reductions in translation rate within the same gene were not covered by this analysis.

#### Gene ontology analyses

Gene ontology analyses were performed using the web application BABELOMICS (http://www.babelomics.org/), using the FatiGO functional enrichment program [38]. All of the gene ontology analyses considered biological function, molecular function, cellular component and transcription factors. The classification was considered significant if the p-value (adjusted for multiple testing) was less than 0.05.

## Supporting Information

Table S1 This table contains the RefSeqs of the human genes with the most significant RTS clusters (p≤0.001). The starting position (in codons) of the cluster (27 codons in length) are shown in column 2.
(0.04 MB PDF)
