# Supplementary material for: Clustering of Codons with Rare Cognate tRNAs in Human Genes Suggests an Extra Level of Expression Regulation
Source: PLoS Genet. 2009 Jul 3;5(7):e1000548. doi: 10.1371/journal.pgen.1000548 (PMC2697378; doi:10.1371/journal.pgen.1000548)
Supplement: Table S1 — This table contains the RefSeqs of the human genes with the most significant RTS clusters (p≤0.001). The starting position (in codons) of the cluster (27 codons in length) are shown in column 2. (0.04 MB PDF) [file pgen.1000548.s001.pdf]

RefSeq	RTS cluster start position	Gene length (codons)	P-value
NM_001678	27	289	< 0.001
NM_017797	27	524	< 0.001
NM_001452	27	443	< 0.001
NM_015156	36	481	< 0.001
NM_024052	36	299	< 0.001
NM_006950	36	704	< 0.001
NM_181718	54	389	< 0.001
NM_001664	63	192	< 0.001
NM_001949	63	464	< 0.001
NM_032273	81	194	< 0.001
NM_033067	81	341	< 0.001
NM_006168	108	366	< 0.001
NM_019048	117	642	< 0.001
NM_006121	117	643	< 0.001
NM_001010926	126	165	< 0.001
NM_014213	153	341	< 0.001
NM_017588	171	333	< 0.001
NM_003830	180	550	< 0.001
NM_152453	225	287	< 0.001
NM_152557	234	643	< 0.001
NM_005027	234	727	< 0.001
NM_003185	243	1084	< 0.001
NM_021954	243	434	< 0.001
NM_024832	261	984	< 0.001
NM_000684	270	476	< 0.001
NM_032421	315	1010	< 0.001
NM_013333	324	549	< 0.001
NM_003108	324	440	< 0.001
NM_021619	333	366	< 0.001
NM_020190	360	405	< 0.001
NM_001099857	360	418	< 0.001
NM_024046	378	500	< 0.001
NM_133375	441	970	< 0.001
NM_016120	450	623	< 0.001
NM_145345	450	486	< 0.001
NM_173354	468	782	< 0.001
NM_021228	567	1311	< 0.001
NM_020695	567	1220	< 0.001
NM_002687	585	716	< 0.001
NM_024682	594	647	< 0.001
NM_004586	603	739	< 0.001
NM_182924	648	903	< 0.001
NM_021235	702	863	< 0.001
NM_013981	711	843	< 0.001

NM_020845	855	1348	< 0.001
NM_018849	945	1285	< 0.001
NM_005392	972	1095	< 0.001
NM_014712	1386	1706	< 0.001
NM_203447	1449	2030	< 0.001
NM_000489	1899	2491	< 0.001
NM_032195	1953	2302	< 0.001
NM_014615	9	1216	< 0.001
NM_025058	72	758	< 0.001
NM_000236	198	498	< 0.001
NM_134269	360	914	< 0.001
NM_006372	441	622	< 0.001
NM_145265	0	259	< 0.001
NM_018638	63	451	< 0.001
NM_080388	63	102	< 0.001
NM_002851	1404	2314	< 0.001
NM_139057	54	1094	< 0.001
NM_015696	99	186	< 0.001
NM_001035518	2349	2385	< 0.001
NM_006150	486	614	< 0.001
NM_001010848	126	695	< 0.001
NM_018963	324	2319	< 0.001
NM_022454	261	413	< 0.001
NM_001008701	414	1473	< 0.001
NM_014109	252	1389	< 0.001
NM_002277	378	415	< 0.001
NM_022093	1035	1298	< 0.001
NM_014333	324	441	< 0.001
NM_017615	18	384	< 0.001
NM_000501	405	723	< 0.001
NM_024693	0	302	< 0.001
NM_002911	927	1117	< 0.001
NM_024589	153	286	< 0.001
NM_003557	0	548	< 0.001
NM_002917	27	330	< 0.001
NM_001232	369	398	< 0.001
NM_003695	99	127	< 0.001
NM_182487	351	651	< 0.001
NM_138983	27	254	0.001
NM_018419	36	383	0.001
NM_032029	63	264	0.001
NM_152710	99	350	0.001
NM_012068	117	281	0.001
NM_173484	126	388	0.001
NM_138415	135	530	0.001
NM_001002918	153	310	0.001

NM_024339	225	340	0.001
NM_016024	288	321	0.001
NM_018330	360	455	0.001
NM_007056	369	673	0.001
NM_003039	369	500	0.001
NM_203504	378	448	0.001
NM_018704	396	638	0.001
NM_178537	459	1038	0.001
NM_015238	522	1112	0.001
NM_020119	567	901	0.001
NM_005641	567	676	0.001
NM_017558	576	1016	0.001
NM_005937	576	1092	0.001
NM_019043	594	665	0.001
NM_014847	729	1086	0.001
NM_000932	918	1233	0.001
NM_032538	1224	1320	0.001
NM_147193	72	619	0.001
NM_024063	81	752	0.001
NM_000217	162	494	0.001
NM_005156	360	551	0.001
NM_005103	27	391	0.001
NM_181809	45	401	0.001
NM_032549	108	174	0.001
NM_147191	117	568	0.001
NM_031923	819	928	0.001
NM_006902	180	216	0.001
NM_020121	468	1515	0.001
NM_022827	18	801	0.001
NM_015012	135	290	0.001
NM_014345	54	2278	0.001
NM_002152	234	698	0.001
NM_014377	198	620	0.001
NM_144625	432	633	0.001
